# Phenotypic and Genomic Profiling of *Escherichia coli* from Irish Raw Milk and Raw Milk Products: A Baseline Study

**DOI:** 10.3390/antibiotics15040326

**Published:** 2026-03-24

**Authors:** Deirdre M. Prendergast, Marian Teeling, Daniel Kelly, Saibh Healy, Gillian Madigan, Sinéad Murphy, Amalia Naranjo-Lucena, Montserrat Gutierrez

**Affiliations:** Department of Agriculture, Food and the Marine (DAFM) Laboratories, Backweston Laboratory Campus, Celbridge, W23 X3PH Kildare, Ireland; marian.teeling@agriculture.gov.ie (M.T.); saibh.healy@agriculture.gov.ie (S.H.); gillian.madigan@agriculture.gov.ie (G.M.); mm.gutierrez@agriculture.gov.ie (M.G.)

**Keywords:** *Escherichia coli*, antimicrobial resistance, raw milk, dairy products, multidrug resistance, ESBL, AmpC, fluoroquinolone, whole genome sequencing

## Abstract

**Background/Objectives:** *Escherichia coli* is a ubiquitous commensal organism in humans, animals, and the environment, but certain strains harbour virulence and antimicrobial resistance (AMR) determinants that can cause significant disease. Food-producing animals, including dairy cattle, may act as reservoirs for AMR *E. coli*, and raw milk and raw milk products can serve as potential exposure pathways to humans. However, data on the prevalence and genomic characteristics of AMR *E. coli* in raw milk in Ireland are limited. This study aimed to describe the occurrence of commensal and clinically relevant AMR *E. coli* in raw milk and raw milk dairy products in Ireland and to characterise their antimicrobial susceptibility and genetic characteristics. **Methods:** A total of 139 raw milk and raw milk dairy product samples were collected and analysed for commensal *E. coli* and fluoroquinolone-resistant, extended-spectrum β-lactamase (ESBL)/AmpC β-lactamase and carbapenemase-producing *E. coli*. AMR patterns were determined in line with EU surveillance guidelines based on the European Committee on Antimicrobial Susceptibility Testing (EUCAST) guidelines which use minimum inhibitory concentration (MIC) breakpoints. Whole genome sequencing (WGS) was conducted on selected isolates to identify AMR genes (ARG), virulence factors, plasmid replicons, efflux pump, disinfectant resistance genes, multi-locus sequence types (MLSTs) and phylogenetic diversity. **Results:** A total of forty-seven *E. coli* isolates were recovered (33.8% isolation rate). Thirteen isolates exhibited resistance to between two and nine antimicrobials, with twelve classified as multidrug resistant (MDR). The highest resistance frequencies were to ampicillin, sulfamethoxazole, trimethoprim and tetracycline. Four fluoroquinolone-resistant isolates, one ESBL producer (*bla_CTX-M-3_*), and one carrying a AmpC promoter mutation were identified; no carbapenemase producers were detected. WGS revealed diverse sequence types, multiple virulence determinants, plasmid replicons, intrinsic efflux pump genes, and limited presence of the disinfectant resistance gene *qacEΔ1*. **Conclusions:** Raw milk and raw milk dairy products in Ireland can harbour AMR *E. coli*, including MDR and potentially pathogenic strains, highlighting the need for ongoing surveillance within the dairy supply chain.

## 1. Introduction

Most *E. coli* strains are ubiquitous commensal organisms, existing harmlessly in humans, animals and their environment and seldom causing disease [1]. However, some strains can be pathogenic due to the presence of specific virulence factors encoded by genes located on the chromosome or mobile genetic elements [1,2]. These pathogens cause a wide range of conditions, from common illnesses like cystitis, urinary tract infections, and gastroenteritis to life-threatening extraintestinal infections, including pneumonia, neonatal meningitis, haemorrhagic colitis or haemolytic uremic syndrome [3]. Genes encoding virulence factors confer the potential for mechanisms to cause disease such as adhesion, invasion and toxin production, amongst others [2,4,5].

Illnesses caused by pathogenic *E. coli* require treatment using antibiotics, including last-resort antibiotics on those specific cases where all other treatment options have failed [6,7]. Resistance to antimicrobials, classified by the World Health Organisation (WHO) as critically important antimicrobials (CIAs) for human medicine, is of major concern since infections caused by these organisms are associated with the most adverse health consequences [8]. Extended spectrum β-lactamases (ESBLs) such as SHV, TEM, CTX-M and AmpC β-lactamases, are enzymes that confer resistance to the CIA cephalosporins and have been commonly identified in *E. coli* from food and food-producing animals in Ireland, Europe and worldwide [9,10,11,12]. The spread of multidrug resistant (MDR) or ESBL-producing *E. coli* due to the misuse and/or overuse of antibiotics in animal and human health as well as the environment is a serious concern [13].

Food-producing animals act as potential reservoirs of antimicrobial resistant (AMR) bacteria and antimicrobial resistant genes (ARGs), which can be transmitted to other animals and humans through direct contact, the food chain or the environment [14,15,16,17,18,19]. This poses a significant public health threat requiring a One Health approach linking human health, animal health and the environment with a strong focus on the emergence and spread of AMR and ARGs [14,16,17]. AMR can be intrinsic, often due to the bacterial cell wall structure, permeability, and the presence of natural efflux pumps that prevent antimicrobial agents from reaching their targets within the cell due to their ability to transport and expel a wide range of unwanted material from the bacterial cell [20,21,22,23]. AMR can also be acquired, due to natural selection, through mechanisms such as horizontal gene transfer driven by plasmids or due to genomic point mutations [24,25]. Bacteria usually acquire ARGs due to selective pressure exerted through the use and misuse of antimicrobials, resulting in them becoming ineffective [13,26]. The use of heavy metals (e.g., copper and zinc) and biocides (e.g., acetic acid and hydrogen peroxide) in food production settings is also known to lead to co-resistance (resistance to several drugs which are linked genetically) through co-selection and co-transfer mechanisms [27,28]. In recent years, especially since the COVID-19 pandemic, non-corrosive biocides such as quaternary ammonium compounds (QACs) are the most frequently used disinfectants in the food industry, with a significant increase in their use [29,30]. Benzalkonium chloride (BAC) and dialkyldimethylammonium chloride (DDAC) are commonly used QACs in the dairy industry where they are used to disinfect udders, milking equipment, milk storage tanks and dairy processing machines [31]. However, it has been demonstrated that their overuse can lead to mutation, toxicity, and the spread of ARG, and can therefore lead to increased AMR in bacteria [29,32].

Dairy cows account for a large proportion of Ireland’s cattle herd, with over 1.4 million recorded in December 2024 [33]. Mastitis, which is the inflammation of the mammary gland, is the most prevalent dairy cow disease worldwide and the main cause of antimicrobial use in the dairy industry [34]. *E. coli* has been reported to be one of the main pathogens responsible for mastitis [34,35,36]. While CIAs should not be used as a first line of treatment, if milk culture results indicate that there is no alternative treatment or in exceptional circumstances, fluoroquinolones, macrolides and third and fourth generation cephalosporins may be used [37]. In addition to that, and prior to the introduction of the European Veterinary Medicines Regulation 2022 [38], treatment of the entire herd with intramammary, long-acting antimicrobials at the end of lactation and during the dry period was carried out in the majority of Irish herds as a preventative measure [39,40].

According to the National Dairy Council, milk for sale in Ireland is generally pasteurised; however, it is legal to sell raw milk and there is a small sector of the population that consumes raw milk and raw milk dairy products such as butter, cheese and cream [41]. Consumers could potentially acquire AMR and ARGs by consuming raw milk or raw milk dairy products [42,43]. Although there is limited information on the occurrence of AMR *E. coli* and ARGs in raw milk and raw milk products in Ireland and Europe, AMR *E. coli* have been detected in milk from animals with mastitis and from healthy cows [44,45,46,47]. Therefore, the aim of this study was to carry out a baseline study to investigate the levels of commensal *E. coli* and fluoroquinolone-resistant, ESBL/AmpC β-lactamase and carbapenemase-producing *E. coli* in raw milk and raw milk dairy products in Ireland to help understand the risks of raw milk as a vehicle for AMR *E. coli.* AMR was determined using phenotypic methods and a subset of the strains were characterised using whole genome sequencing (WGS) to obtain a deeper understanding of the ARGs together with other related or significant genetic determinants, i.e., virulence factors, plasmid replicons, disinfectant resistance, efflux pump genes, MLST and phylogenetic diversity.

## 2. Results

### 2.1. Bacterial Culture

Forty-seven *E. coli* isolates were recovered from the 139 milk and dairy product samples tested (33.8% isolation rate), which is summarised in Table 1. The proportion of commensal *E. coli* positive samples amongst the sample types was 25.3%, 36.8% and 35.7% from raw milk collected as part of the national residue control plan of Ireland (RM-NRCP), raw milk retailed as raw milk (RM-FBO), and raw milk dairy products (RDP), respectively. Although a higher proportion of commensal *E. coli* were recovered from RM-FBO samples compared to RM-NRCP, chi-squared analysis indicated that the difference was not significant (*p* > 0.05). In addition, four isolates were recovered from the MacConkey agar plates with the ciprofloxacin paper disc (three RM-NRCP and one RDP from cream), and two isolates (RM-FBO and RM-NRCP) were isolated from MacConkey plates with cefotaxime. No presumptive carbapenemase-producing *E. coli* were isolated from any of the samples tested.

### 2.2. Antimicrobial Susceptibility Testing

AST resulted in 34 isolates being found fully susceptible to all antimicrobials tested and 13 isolates resistant to between two and nine antimicrobials (Table 2). Although a higher proportion of resistant *E. coli* were recovered from RM-NRCP samples compared to RM-FBO, chi-squared analysis indicated that the difference was not significant (*p* > 0.05). The majority of resistant isolates (12 out of 13) were MDR, i.e., resistant to at least one antimicrobial in three or more antimicrobial classes consistent with established conventions, as defined by their pharmacological class and mechanism of action, e.g., beta-lactams, tetracyclines, sulfonamides, etc. (Table 2). Ampicillin resistance was the highest (25.3%), followed by sulfamethoxazole (23.4%), trimethoprim (21.3%) and tetracycline (14.9%). Resistance levels were moderate for ciprofloxacin, nalidixic acid, and chloramphenicol (8.5%), and only one isolate was resistant to gentamicin (2.1%). None of the isolates were resistant to colistin (polymyxin) or meropenem (carbapenem). Amongst the two presumptive ESBL/AmpC β-lactamase producers, the *E. coli* isolated from RM-NRCP was found to be a presumptive AmpC while the isolate from RM-FBO was found to be a presumptive ESBL producer when the EU Surveillance ESBL EUVSEC2 AST plate was used. The four presumptive fluoroquinolone isolates were indicative of phenotypic AMR chromosomal fluroquinolone resistance i.e., resistant to both ciprofloxacin and nalidixic acid [48].

### 2.3. Whole Genome Sequencing

Following WGS, one of the 33 sequenced genomes failed the quality metrics (average coverage of 40×) from the raw sequencing data and was subsequently excluded from the WGS analysis. This was an isolate from RM-NRCP which was fully susceptible. Over each run on the NextSeq, the index reads were evenly distributed.

**Figure 1:**
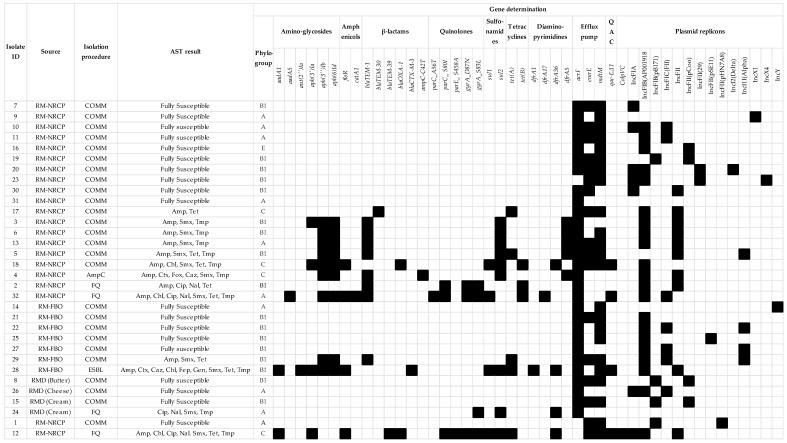
Phenotypic and genomic antimicrobial resistance of *E. coli* isolates, along with genes encoding efflux pumps, quaternary ammonium compounds resistance, and plasmid replicons. RM-NRCP: raw milk collected as part of the national residue control plan of Ireland; RM-FBO: raw milk retailed as raw milk; RMD: raw milk dairy product; COMM: commensal; FQ: fluoroquinolone resistant; Amp: ampicillin; Chl: chloramphenicol; Cip: ciprofloxacin; Nal: nalidixic acid; Smx: sulfamethoxazole; Tet: tetracycline; Tmp: trimethoprim; Ctx: cefotaxime; Fep: cefepime; Gen,: gentamicin; QAC: quaternary ammonium compounds.

The four presumptive chromosomal fluoroquinolones were confirmed genotypically and three of the four displayed double mutations in *gyrA* and two in *parC*/*parE* (Figure 1). The most frequent mutations were observed in codon 83 of *gyrA* and codon 80 of the *parC* gene. The presumptive fluoroquinolone *E. coli* recovered from RDP harboured a single mutation in codon 83 of *gyrA* (Figure 1). Although this isolate was classified as resistant to ciprofloxacin and nalidixic acid according to EUCAST epidemiological cut-off values, the presence of a single *gyrA* mutation indicated low-level fluoroquinolone resistance, consistent with the borderline ciprofloxacin MIC of 0.25 mg/L (Supplementary Table S2).

Ampicillin resistance was observed in 12 isolates which included the ESBL and AmpC isolates. The presumptive AmpC β-lactamase producer encoded the upregulated AmpC promotor at position 42 (C-42T mutation), and harboured the *bla_TEM-1_* gene, while the presumptive ESBL producer harboured the gene *bla_CTX-M-3_* (Figure 1). *Bla_TEM-1_* was identified in seven of the rest of the ampicillin-resistant *E. coli* isolates, while *bla_TEM-30_* and *bla_TEM-39_* in combination with *bla_OXA-1_* and *bla_OXA-1_* were identified in the other three ampicillin-resistant *E. coli* isolates. Finally, four isolates resistant to chloramphenicol harboured the *floR* gene, eight isolates resistant to tetracyclines the *tet(A)* or *tet(B)* genes, ten isolates resistant to both trimethoprim and sulfamethoxazole different variants of the *dfrA* and *sul1*/*sul2* genes and one isolate the gentamicin resistance gene, *ant(2″)Ia* (Figure 1). Other genes known to confer resistance to aminoglycosides were observed in nine isolates, namely *aadA1*, *aadA5*, *aph(3′)Ia*, *aph(3″)Ib* and *aph(6)Id*, although these genes confer resistance to antimicrobials not included in the EUVSEC panels used here, so phenotypic confirmation for those determinants was not available.

Efflux pump genes were present in all 32 *E. coli* genomes, i.e., both fully susceptible and AMR isolates. Three efflux pump genes were identified, i.e., the *acrF*, *mdtM* and *emrE* genes, either alone or in combination in 29, 23 and 17 of the genomes, respectively (Figure 1). 

The truncated gene *qacEΔ1* (X68232; partial match with 84.7% coverage and 100% identity) was identified in four of the sequenced *E. coli* isolates (Figure 1). Three of these isolates were from RM-NRCP and two of these were fluoroquinolone resistant. The ESBL isolate from RM-FBO also harboured the *qacEΔ1* gene. In each case, the *qacΔ*-like sequence was located on the same contig as *sul1*, and in three isolates it co-occurred with additional ARGs with cassette-associated arrangements, i.e., *aadA1*/*aadA5*, *dfrA17*, *catA1*, *ant(2″)-Ia*, and/or *bla_OXA-1_*.

Fifteen plasmid replicons were identified in 30 *E. coli* isolates: IncFIB(AP001918), IncFII, IncFIA, IncFIC(FII), IncFIB(pB171), IncI1-I(Alpha), IncFII(pCoo), IncFII(29), IncX1, IncFII(pSE11), IncFII(pHN7A8), IncY, IncI2(Delta), ColpVC and IncX4 present respectively in 21, ten, seven, seven, four, four, four, two, and one of each of the rest of the isolates. Eighteen of the 19 fully susceptible and 12 the 13 AMR isolates harboured at least one plasmid replicon. There were 14 plasmid replicons identified amongst the fully susceptible isolates and six among the AMR isolates, with IncFIB(AP001918) and IncFII identified in 85% and 69% of resistant *E. coli* and only 52% and 5% of the fully susceptible isolates. The only resistant isolate without detectable plasmid replicons was the fluoroquinolone-resistant *E. coli* isolate recovered from RMD.

In silico phylogrouping permitted the classification of *E. coli* isolates into four of the seven main phylogroups, i.e., A, B1, C and E, in ten, seventeen, four and one isolate(s), respectively. Two of the fluoroquinolone-resistant isolates were assigned to phylogroup A, whereas the other two were assigned into phylogroups B1 and C. The ESBL- and AmpC-producing isolates were assigned to phylogroups B1 and C, respectively (Figure 1). There was 100% concordance between the in silico PCR assay and the Mash genome-clustering tool.

**Figure 2:**
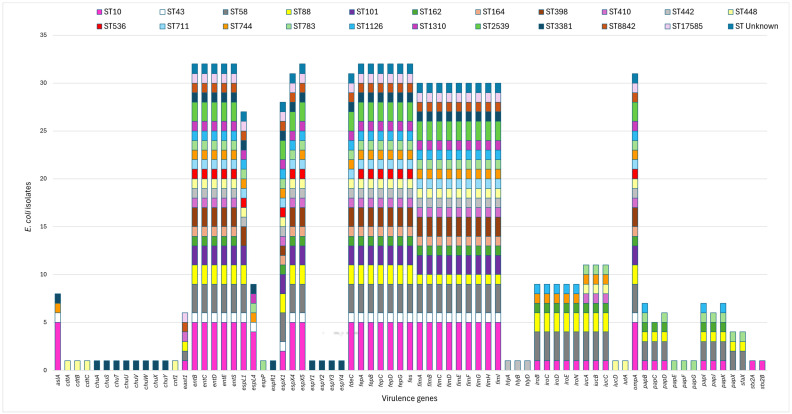
Distribution of virulence genes and *E. coli* sequence types amongst the 32 sequenced isolates.

In silico MLST typing yielded 22 different STs amongst the 32 *E. coli*, with ST10 the most common followed by ST58 identified in five and three isolates respectively. One isolate of unknown ST was also identified. The distribution of virulence genes amongst the 22 STs of *E. coli* isolates is shown in Figure 2. Major virulence determinants were identified in the sequenced *E. coli* isolates, including Shiga toxin-producing (*stx2A* and *stx2B*), the cytolethal distending toxin operon (*cdtABC*), α-hemolysin (*hlyA*, *hlyB*, *hlyD*), the gene encoding arylsulfatase A enzyme (*aslA*), cytotoxic necrotizing factor 1 (*cnf1*), EAST1 (*astA*/*east1*), high-affinity iron-acquisition systems (*entB*–*E*, *entS*, *fepA*–*D/G*, *fes*), iron uptake including the salmochelin siderophore system (*iroBCDEN*), the 8-gene cluster (*chu* genes) involved in heme transport and processing (*chuA*, *chuS-Y*), and the type III secretion system (T3SS) effector genes (*espL1*, *espL4*, *espP*, *espR1*, *espX1*, *espX4*, *espX5*, *espY1*, *espY2*, *espY3*, *espY4*). Multiple *fep* genes encoding ferric enterobactin transport functions (*fepA*, *fepB*, *fepC*, *fepD*, *fepG*) were identified in all but one of the *E. coli* isolates, as was the *ompA* gene which affects adhesion and plays a key role in the resistance of bacteria to antibiotics. Genes involved in aerobactin biosynthesis (*iucA*–*D*, *iutA*), the outer membrane hemin receptor for heme uptake (*chuA*–*Y*), and key adherence factors such as *fdeC*, type 1 fimbriae (*fimA*–*H*), P fimbriae (*papA*–*K*, *papG*), *sfaX* and associated regulators were also identified. In general, all isolates within each ST displayed the same virulence profile. Amongst all 32 isolates, one isolate of ST10 (identified as fluoroquinolone resistant), harboured two stx2 gene toxins (*stx2A* and *stx2B*). The aggregative heat-stable enterotoxin 1 (EAST1) was identified in six isolates of ST10, ST58, ST88, ST410, ST8842 and ST17585 (Figure 2).

**Figure 3:**
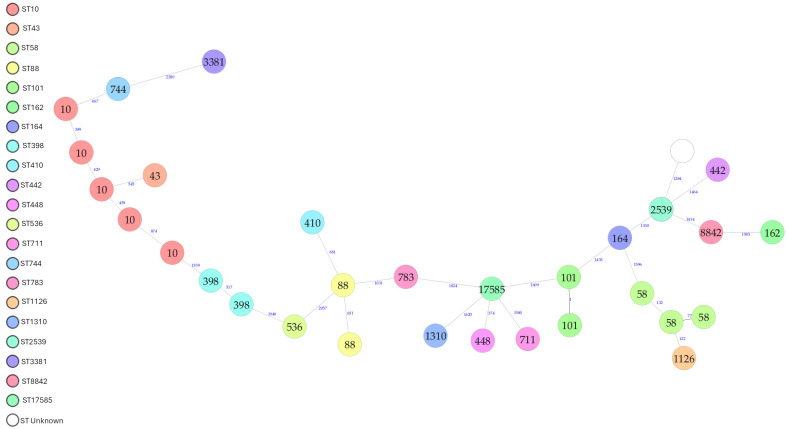
MST for 32 *E. coli* isolates based on 2528 columns, pairwise ignoring missing values, logarithmic scale. Each coloured circle refers to the MLST (Warwick scheme) for a single strain and the numbers within each circle refer to the MLST for each isolate. A line within a circle refers to two isolates with the same ST. The numbers between nodes indicate the number of allelic differences (cgMLST).

The minimum spanning tree (MST) showed a wide range of diversity with 30 different cgMLSTs observed amongst the 32 *E. coli* isolates (Figure 3). Two clusters were identified, one of ST101 which included two isolates that were one allele different through cgMLST, and both were isolated from RM-FBO and both fully susceptible. The second cluster included two isolates of ST2539 isolated from RMD; both were indistinguishable to each other, and one was isolated from butter and the other from cream (Figure 3).

## 3. Discussion

Although raw milk and raw milk products are appealing to some consumers as a nutritious alternative to heat-treated products, the lack of a thermal treatment poses a risk. Raw milk is not included in the AMR EU surveillance programme under Commission Implementing Decision (EU) 2020/1729 [49], and to the authors’ knowledge, no previous work has examined AMR, virulence factors, plasmid replicons or disinfectant-related genes in *E. coli* from Irish raw milk. Only limited food safety data exist from the past two decades for raw milk in Ireland; notably, our group reported Shiga toxin-producing *E. coli* (STEC) in 14% of raw milk filters and 1% of raw milk in 2017–2019 [50]. In addition, the Food Safety Authority of Ireland (FSAI) reported STEC in 6% of raw milk filters in 2012–2013 [51], while a study conducted between 2001 and 2003 detected *E. coli* O157 in 12.5% of milk filters sampled [52].

The consumption of raw milk and raw milk cheese in Europe represents a recognised public health concern due to frequent contamination with *E. coli*, including STEC and AMR strains [53,54]. European studies consistently report *E. coli* in raw milk at variable but often significant levels (approximately 2–22%), while raw milk cheese, particularly artisanal products, may show even higher levels. Importantly, a substantial proportion of isolates display AMR, with MDR phenotypes reported in 27–40% of strains which are commonly resistant to β-lactams, tetracyclines, aminoglycosides, and fluoroquinolones [55,56,57,58]. The repeated detection of virulent and resistant *E. coli* in unpasteurised dairy products, together with documented STEC outbreaks linked to raw milk cheese in several European countries, underscores the dual risk of foodborne infection and dissemination of AMR via the raw milk food chain [59,60].

Our study identified *E. coli* that were MDR, which contained virulence-, pathogenic-, and disinfectant-related genes in raw milk and raw milk products. This reinforces the need for maintaining high farm hygiene measures, as *E. coli* levels in milk are known to reflect the cleaning efficacy of milking equipment and bulk milk storage temperatures [61,62]. Approximately one fourth of the *E. coli* isolates in our study (27.7%) exhibited AMR and 25.5% were classified as MDR. To the best of the authors’ knowledge, there is no previous research on AMR in raw milk and their products in Ireland. While the sampling framework does not permit inference to the wider raw milk population, these findings provide a useful descriptive reference point for future investigations in Ireland. Encouragingly, no carbapenemase-producing *E. coli* were detected. The lower proportion of resistant isolates in raw milk destined for direct consumption, compared with milk intended for pasteurisation, may reflect greater compliance with the relevant hygiene requirements laid down in Annexes I and II of Regulation (EC) 852/2004 [63], as well as those set out in Annex III (Section IX) of Regulation (EC) No 853/2004 [64].

The sequencing results of this study identified a variety of resistance determinants in a subset of the isolates. Efflux pump genes *acrF*, *mdtM*, and *emrE* were detected either alone or in combination in all 32 isolates. These genes are recognised components of the intrinsic *E. coli* genome, and their presence is therefore expected in both susceptible and resistant strains. Consequently, gene detection alone is not discriminatory for AMR phenotypes. *AcrF*, for which encoding genes were found in all but three of the sequenced isolates, is a member of the resistance nodulation and cell division (RND) family and represents one of the seven known RND transporters in *E. coli*. Through the tripartite efflux AcrEF-TolC system [65], it contributes to the extrusion of a broad array of hydrophobic and amphiphilic compounds, including a wide range of antimicrobials and biocides, although non-functional variants have also been described [66]. Genes encoding *EmrE*, which extrudes hydrophobic compounds [61,67] and belongs to the small multidrug resistant (SMR) family, and *mdtM*, a major facilitator superfamily (MFS) antiporter that actively expels neutral and cationic compounds including QACs, were also found in more than half of the isolates.

Previously reported research showed that efflux pump mechanisms are widespread in *E. coli* but do not necessarily confer QAC tolerance unless induced under specific conditions [62,65]. Moreover, recent experimental work demonstrated that *E. coli* may develop increased tolerance to benzalkonium chloride through adaptive mechanisms independent of integron-encoded qac genes [68]. In the present study, a *qacΔ*-like sequence was detected with incomplete coverage (84.7% coverage, 100% identity to X68232) in four isolates. Notably, in all cases this sequence was co-located with *sul1*, and frequently with additional AMR genes including *aadA1*/*aadA5*, *dfrA17*, *catA1*, *ant (2″)-Ia*, and *bla_OXA-1_*. This conserved gene arrangement is consistent with class 1 integron-associated regions, where *sul1* and truncated qac-associated sequences are commonly observed alongside cassette-borne resistance determinants. The incomplete coverage of the *qacΔ* hit supports the interpretation that this likely represents a truncated *qacEΔ1*-like sequence rather than an intact functional determinant. Importantly, while this genomic architecture suggests the potential for co-selection and co-dissemination of integron-associated resistance regions under antimicrobial pressure, the presence of qac-associated sequences alone cannot be interpreted as evidence of phenotypic disinfectant resistance.

Our findings highlight the need for follow-up phenotypic assays, for example QAC-specific MIC testing, time-kill studies, and biofilm tolerance assessment investigations on the *E. coli* isolates carrying *qacEΔ1*, to determine whether this gene contributes to measurable reductions in QAC susceptibility. This is because although *qacEΔ1* is frequently embedded within class 1 integrons, which are mobile genetic elements (MGE), its association with phenotypic QAC tolerance has often been reported to be weak and inconsistent [69,70,71]. Genetic detection alone cannot reliably predict disinfectant resistance, underscoring the importance of phenotypic validation [20,72,73]. Genes conferring resistance to other biocides, such as chlorhexidine, hydrogen peroxide, ethanol, isopropanol, peracetic acid, povidone-iodine and sodium hypochlorite [74] were not identified in the present study.

The widespread detection of plasmid replicon sequences in both resistant and fully susceptible *E. coli* indicates that plasmid-associated elements are common in dairy-associated commensal populations and are not solely driven by AMR [75]. The high proportion of plasmid replicons in both resistant and susceptible isolates, particularly IncF plasmids such as IncFIB (AP001918) and IncFII, suggests that these lineages frequently harbour sequences that are homologous to known plasmid incompatibility groups. However, replicon detection alone does not permit the reconstruction of complete plasmids nor confirm plasmid localization of ARGs [75,76,77]. IncF plasmids are widely recognised as important contributors to horizontal gene transfer in *E. coli* [78]. Notably, IncFIB (AP001918), which is frequently reported in association with ESBL-producing *E. coli* in human infections [79,80], was also detected in a substantial proportion of fully susceptible isolates. IncL/M plasmid replicons were not detected. PlasmidFinder, which relies on a precompiled database, may fail to detect some plasmids, particularly larger plasmids >50 kb [81]. The main limitation for plasmid characterisation in this study is the use of short-read sequencing, which can hinder accurate reconstruction of plasmid structures, especially when repetitive elements or regions shared with the chromosome are present, preventing full characterisation of complete plasmid sequences [81,82].

This observation should be interpreted cautiously, as replicon presence does not demonstrate that isolates carry intact plasmids comparable to those described in clinical settings, nor that resistance determinants are plasmid-borne.

Chromosomal fluoroquinolone resistance was confirmed through mutations in *gyrA* and *parC*, which reduce susceptibility and can increase efflux pump expression [83,84]. The fluoroquinolone resistance observed in the four *E. coli* isolates aligns with the expected sequential acquisition of quinolone-resistant determining region (QRDR) mutations. Three isolates carried multiple mutations in *gyrA* and/or *parC*, consistent with higher-level resistance, while one isolate had only a single *gyrA* Ser83Leu mutation and a borderline ciprofloxacin MIC, reflecting low-level resistance. This pattern demonstrates that even minimal mutations can confer detectable resistance and may gradually accumulate to produce higher-level resistance.

The co-occurrence of chromosomal AMR determinants, plasmid replicons, virulence factors, and biocide-resistance genes in raw milk isolates reflect strong and overlapping selective pressures within dairy production environments [85,86]. Antimicrobial use in dairy cattle, particularly for the treatment of mastitis, can enrich intrinsic resistance and efflux-mediated tolerance, contributing to the selection of resistant bacterial populations [87]. Manure and slurry can further disseminate resistant bacteria and antimicrobial residues, sustaining environmental selection and contaminating soil and water [88,89,90]. Routine exposure to biocides, heavy metals, and other environmental contaminants can promote co-selection through efflux activation and membrane adaptation [27]. Environmental pathways, including contaminated water, biofilms on milking surfaces, and movement of animals or personnel, facilitate ongoing circulation and persistence of resistant strains [91]. Collectively, these pressures allow chromosomal resistance traits to persist and spread independently of plasmid-mediated exchange, highlighting the need for an integrated approach towards antimicrobial use, rigid hygiene measures at both farm and milk processing levels, and environmental management practices given that soil antibiotic resistomes are widespread and connected to human and environmental AMR reservoirs [92,93,94].

The present study revealed ST10 to be the most prevalent lineage, with all ST10 isolates belonging to phylogroup A, a human-associated lineage that has been linked to the dissemination of AMR via plasmids and other mobile genetic elements [75]. ST58 was the second most common and belonged to phylogroup B1, an emerging zoonotic lineage increasingly associated with MDR, ESBL production, and extraintestinal infections [77,79,80,95]. Among the isolates, one ST10 was MDR and fluoroquinolone-resistant, while all three ST58 isolates were resistant to at least three antimicrobial classes. Apart from one fluoroquinolone isolate (ST10) that was recovered from cream, all remaining ST10 and ST58 isolates were obtained from raw milk, indicating that these lineages may serve as vehicles for resistance determinants along the dairy supply chain [77,79,95]. Additional resistant lineages included an AmpC β-lactamase-producing ST88 isolate of phylogroup C, an ESBL-producing ST1126 of phylogroup B1, and three more fluoroquinolone-resistant isolates within phylogroups C (two isolates), and B1, demonstrating that AMR in raw milk-associated *E. coli* occurs across multiple phylogenetic backgrounds [77,79,80,95]. While no direct evidence of transmission to humans was assessed in this study, the presence of potentially high-risk lineages highlights the importance of continued surveillance of the dairy food chain within a One Health framework, encompassing human, animal, and environmental reservoirs to better understand and mitigate dissemination of strains of public health relevance.

Amongst the virulence genes identified, some were associated with functions including stress, survival, regulation, iron uptake, type-three secretion systems, invasion, adherence, colonisation and toxin production. The identification of major virulence determinants in the sequenced *E. coli* isolates highlights virulence profiles with public health relevance. Toxins such as Shiga toxin are strongly linked to haemorrhagic colitis and haemolytic uremic syndrome, representing one of the most critical food and waterborne threats [96,97]. Likewise, *cdtABC*, *hlyA*, and *cnf1* can contribute to epithelial damage, systemic inflammation, and severe extraintestinal infections, including urosepsis and neonatal meningitis [98,99,100], while it has also recently been reported that *cnf1* promotes colorectal carcinogenesis [101]. Iron-acquisition systems including enterobactin, salmochelin, aerobactin, and heme-uptake pathways are key fitness determinants in host environments and are strongly associated with high-risk extraintestinal pathogenic *E. coli* lineages [102]. Adherence factors type 1 and P fimbriae enhance colonisation of the urinary and gastrointestinal tracts and facilitate progression to invasive disease [103,104]. In this study, 30 isolates carried the *fimA* to *fimH* type 1 fimbrial genes, which encode adhesin proteins that are key contributors to biofilm formation and bacterial adherence [105]. The coexistence of these elements may enhance the ability of strains to persist in the environment, adapt to different hosts and contribute to the spread of AMR, highlighting the interconnected risks across human, animal and environmental health. This co-existence could not only limit therapeutic options but also increase the likelihood of horizontal gene transfer, environmental persistence, and foodborne transmission, underscoring the need for a comprehensive, up-to-date surveillance across the Irish dairy sector [106].

A limitation of this baseline study was that the quantitative burden of *E. coli* in raw milk and raw milk dairy samples was not assessed. The presence/absence approach was taken because individual colonies recovered from a single sample may represent genetically distinct strains with different AMR profiles, and it cannot be assumed that all the colonies present share the same AMR phenotype or genotype. As strain heterogeneity was anticipated, analysing multiple colonies per sample was not undertaken, as this would still not reliably represent the dominant strain population or allow meaningful extrapolation for exposure relevance. Future work incorporating quantitative enumeration alongside characterisation of multiple isolates per sample, or culture-independent approaches such as metagenomic analysis, would enable improved assessment of strain diversity and the magnitude of AMR exposure associated with raw milk products, thereby strengthening risk assessment frameworks.

## 4. Materials and Methods

### 4.1. Sample Selection

A total of 139 samples received in our laboratories between March and September 2022 as part of different routine DAFM testing programmes were selected for this study: (A) 87 raw milk samples from individual dairy farms collected as part of the national residue control plan of Ireland (RM-NRCP). This milk was intended for pasteurisation or further processing. (B) 38 raw milk samples from individual food business operators (RM-FBOs) approved for selling raw milk for human consumption. This milk was intended to be consumed raw. (C) 14 raw dairy products (RDP) intended for human consumption: five butter, seven cheese, and two cream samples.

### 4.2. Isolation of ESBL-/AmpC β-Lactamase- and Carbapenemase-Producing E. coli

Samples were diluted 1:10 in buffered peptone water (BPW; Syntec Scientific, Dublin, Ireland) and incubated for 18–22 h at 37 °C. ESBL-, AmpC-, and carbapenemase-producing *E. coli* were isolated using the method previously described and recommended by the European Union Reference Laboratory for Antimicrobial Resistance (EURL-AR) [107]. In brief, the enriched BPW was spread and plated onto appropriate selective agars, i.e., MacConkey plates supplemented with 1 mg/L cefotaxime (E&O laboratories Ltd., Bonnybridge, UK) for ESBL- and AmpC-producing *E. coli* and ChromID CARBA and ChromID OXA-48 plates (BioMérieux, Basingstoke, UK) for the selective isolation of carbapenemase-producing *E. coli*. All selective plates were incubated at 37 °C for 18–22 h. Smooth, round, bright pink to red, non-mucoid colonies with a darker pink zone of precipitated bile salts on MacConkey and CTX were considered presumptive ESBL/AmpC producers, while smooth, round, well-defined pink to dark pink colonies on chromID^®^ CARBA and OXA-48 plates were considered presumptive carbapenemase producers. All selective plates were incubated at 37 °C for 18–22 h. Presumptive colonies on the chromogenic agars and those that grew close to the ciprofloxacin disc were purified on the selective media from which they were obtained and on cystine–lactose–electrolyte-deficient (CLED) agar (W12515, Fannin Ltd., Dublin, Ireland) as previously described [9].

### 4.3. Isolation of Fluoroquinolone-Resistant E. coli

Isolation of fluoroquinolone-resistant *E. coli* was carried out according to the method previously described [9]. In brief, a sterile cotton swab was dipped into enriched BPW and spread over the entire surface of a MacConkey agar plate (E&O laboratories) which was streaked with the same swab after rotating the plate approximately 60 degrees. Immediately after the plate was inoculated, a 5 µg ciprofloxacin paper disc (CT0425B, Fannin Ltd.) was placed aseptically onto the middle of the agar plate. Plates were incubated as described above and presumptive colonies were those that grew close to the ciprofloxacin disc. Presumptive colonies were purified as previously described [9]. 

### 4.4. Isolation of Commensal E. coli

Commensal *E. coli* was isolated from raw milk and raw milk products following the standard ISO 16649-2:2001 [108]. Milk, cream and butter samples were diluted in maximum recovery diluent (MRD: SKU: KM0040, E&O Laboratories) while cheese samples were diluted in 2% tri-sodium citrate solution (Scientific Laboratory Supplies, Rathcoole, Dublin), as previously described [109]. A 1 mL volume of the required dilutions (milk: 10^0^ and 10^−1^, cream and butter: 10^−1^ and cheese: 10^−1^ and 10^−2^) was pipetted into the centre of a Petri dish followed by the addition of 15 mL Tryptone Bile X-Glucuronide (TBX) Medium (CM0945B, Fisher Scientific Ltd., Dublin, Ireland), which was tempered to 45 ± 2 °C followed by gentle mixing. Once solidified, plates were incubated for 4 h at 37 °C followed by 18 to 24 h at 44 ± 1 °C.

### 4.5. Positive and Negative Controls

For each batch of samples tested, positive and negative controls were used, i.e., *E. coli* NCTC 13353 (ESBL producer), *E. coli* NCTC 13476 (carbapenemase resistant) and *E. cloacae* 03-577qnr-AI (fluoroquinolone resistant). *E. coli* ATCC 25922 was used as the commensal positive control and as a negative control for the ESBL/AmpC β-lactamase producers and carbapenemase-producing *E. coli*. These control isolates were obtained from the EURL-AR.

### 4.6. Bacterial Species Confirmation

Matrix-assisted laser desorption ionisation-time of flight (MALDI-TOF) mass spectrometry (Bruker Daltronics GmbH, Bremen, Germany) was used to confirm all *E. coli* isolates as previously described [110]. As indicated by EURL-AR protocols, three colonies were individually subcultured. If the first was identified as *E. coli*, no other colonies were identified. If the first subculture was not identified as *E. coli,* then the second and third were eventually identified. Confirmed *E. coli* isolates were maintained at −80 °C pending further analyses.

### 4.7. Antimicrobial Susceptibility Testing

Antimicrobial susceptibility testing (AST) was carried out by determining the minimum inhibitory concentration (MIC) using the Sensititre™ EU surveillance *Salmonella*/*E. coli* EUVSEC3 AST Plates (Thermo Scientific, Waltham, MA, USA). The antimicrobials tested using this plate include amikacin, ampicillin, ceftazidime, chloramphenicol, ciprofloxacin, colistin, cefotaxime, gentamycin, meropenem, nalidixic acid, sulfamethoxazole, tetracycline, tigecycline, and trimethoprim. A control strain of *E. coli* ATCC 25922 was included alongside each batch of strains that were tested. In addition, the negative control well was streaked onto blood agar for confirmation of no contamination. Presumptive ESBL-, AmpC- or carbapenemase-producing *E. coli* isolates were tested further with the second panel of antimicrobials, the Sensititre™ EU Surveillance ESBL EUVSEC2 AST plates (Thermo Scientific), which included temocillin, cefoxitin, cefepime, and clavulanate in combination with cefotaxime and ceftazidime, for the detection of ESBL and AmpC production. EUVSEC2 plates also included imipenem, meropenem and ertapenem to phenotypically verify presumptive carbapenemase-producing *E. coli* as defined by EUCAST [111]. Isolates exhibiting resistance to cefotaxime and/or ceftazidime, with a cefepime minimum inhibitory concentration (MIC) > 0.125 mg/L and demonstrating a ≥8-fold reduction in MIC for cefotaxime–clavulanate or ceftazidime–clavulanate compared with cephalosporin alone were classified as ESBL producers. Isolates resistant to cefotaxime and/or ceftazidime that were also resistant to cefoxitin and exhibited no reduction in MIC, or a reduction in fewer than three twofold dilution steps in the presence of clavulanate, were classified as AmpC β-lactamase producers.

Depending on the results obtained from the susceptibility testing performed with the second panel, the isolates were subsequently classified as presumptive ESBL, AmpC, ESBL/AmpC or carbapenemase producers according to EUCAST 2013 criteria [111,112]. Classification of presumptive chromosomal or plasmid mediated fluoroquinolone resistance was carried out as previously described [8].

### 4.8. Whole Genome Sequencing Analysis

#### 4.8.1. Selection of Isolates

In total, 33 isolates were selected for WGS; this included all phenotypical AMR isolates (13) and 20 isolates that were fully susceptible to all antimicrobials.

#### 4.8.2. Culture Preparation and DNA Extraction

*E. coli* isolates were recovered from frozen stocks on Columbia agar and horse blood (E&O Laboratories) and incubated for 24 h at 37 °C. A 1 μL loopful of pure culture was taken from the plate and re-suspended in 100 μL of nuclease free water. DNA was extracted using the MagNA pure 96 system (Roche, Dublin, Ireland) and quality checked as previously described [48].

#### 4.8.3. Library Preparation, Amplification, Denaturing and Sequencing of Libraries

Sample libraries for all isolates were prepared using the Illumina DNA prep kit (Illumina, Inc., Eindhoven, The Netherlands) as per manufacturer’s instructions and using 30 μL of stock DNA to provide an input range of 100–500 ng. The library was amplified by five PCR cycles, with normalisation and denaturing performed as previously described [48]. The molarity of each sample was normalised to 2 nM and the same quantity of each normalised library was pooled to produce a pooled amplified library which was then loaded with 2% PhiX (control library). The isolates included in this study were distributed over four sequencing runs on the NextSeq 2000 (Illumina) using a NextSeq™ 1000/2000 P1 Reagent Cartridge (300 cycles) and NextSeq™ 1000/2000 P1 Flow Cell (Illumina, San Diego, CA, USA).

### 4.9. Data Analysis

#### 4.9.1. Statistical Analysis

Using Epitools [113], a chi-square test based on cross-tabulation of the observed data was performed to explore potential associations between sample submission streams (RM-NRCP vs. RM-FBO) and the recovery of commensal *E. coli* within the study dataset. In addition, Epitools was used to determine potential associations between the % AMR among RM-NRCP and RM-FBO. The analysis was intended solely as a descriptive assessment of association within the sampled submissions and does not support population-level inference. Contingency tables were analysed, and results were considered statistically significant when *p* < 0.05. Due to the low sample numbers of butters, cheese and creams that were received, statistical analysis was not performed on RDP.

#### 4.9.2. Analysis of WGS Data

The following run metrics were used to check that each WGS run met the basic quality metrics requirements for raw sequence data: >85% bases higher than Q30 at 2 × 150 bp and cluster density of 4974.18 k/mm^2^. In addition, the percentage of reads that aligned to the PhiX was also checked to ensure that the starting concentration of the libraries was not over- or under-estimated. Bruker MBioSEQ Ridom Typer software (formerly known as SeqSphere), version 11.1.0 2025-10 (Ridom GmbH, Münster, Germany) was used to process and analyse WGS data, i.e., quality trimming, de novo assembly, quality assessment and analysis of WGS data. Raw reads (FASTQ) were automatically trimmed prior to assembly using the default settings for trimming, i.e., trimmed at both ends until the average base quality was >30 in a window of bases. Paired-end raw read data were assembled using the SPAdes assembler’s algorithm (V3.15.4) set at the automatic mode to determine the coverage cut-off and minimum length of contigs. De novo assemblies with >50x coverage, 5.0 Mb ± 10% bp in size, an N50 ≥ 15,000 and core percentage of ≥95% were included in the downstream WGS analysis. Species confirmation, MLST using the Warwick scheme, and core genome MLST (cgMLST) from assembled *E. coli* genomes was conducted using SeqSphere+. Additionally, the integrated NCBI AMRFinderPlus (AMR Gene Finder plus) software, version 4.0.15 [114,115,116] and virulence factor database [117] (VFDB) were used to identify AMR and virulence, respectively. Furthermore, genes related to reduced susceptibility to disinfectants and general efflux were also identified within AMRFinderPlus. In silico phenotyping was performed using the freely available online ClermonTyper, based on an in silico quadruplex PCR targeting the genes *arpA*, *chuA*, *yjaA* and TspE4.C2 and is accessible at http://clermontyping.iame-research.center/ (accessed on 16 March 2026). Each FASTA file was uploaded, as input and the species/phylogroup were determined in two distinct ways: by in silico PCR and Mash. Mash is the species phylogroup of the nearest genome [118]. The presence or absence of the different amplicons constituted a profile that allowed for phylogroup assignment. A minimum spanning tree (MST) was constructed using MBioSEQ Ridom Typer. CGE tools (Center for Genomic Epidemiology), available at https://www.genomicepidemiology.org/services/ (accessed on 16 March 2026), were used for further analysis of the assembled data, i.e., in silico detection of plasmid replicons using PlasmidFinder 2.1 [79,119], identification of MobileElementFinder 1.0 [120] and PathogenFinder 1.1 [121] were used to predict the potential of each *E. coli* isolate to be a human pathogen by uploading the FASTA file and choosing the proteobacteria as the phylum and gamma as the class. The number and type (pathogen specific or non-pathogen specific) of query strain orthologues in the protein family database are used to assign a human pathogen probability score (range 0.0–1.0), where a probability score > 0.5 is considered as the threshold for consideration as a human pathogen.

## 5. Conclusions

In conclusion, to the best of our knowledge, this is the first study to characterise AMR, STs, virulence, plasmid replicons, and disinfectant-related determinants in *E. coli* from raw milk and raw milk dairy products in the Republic of Ireland. Although the overall levels of AMR were moderate, the detection of MDR isolates, ESBL/AmpC producers, high-risk sequence types (ST10, ST58), and Shiga toxin genes indicate that raw milk can act as a vehicle for transmission of clinically significant *E. coli* through the food chain. The identification of isolates harbouring genes encoding for QAC resistance highlights the potential selective effects of biocide use, which is not routinely monitored. Strengthening surveillance efforts, optimising hygiene practices and ensuring prudent use of antibiotics and biocides are therefore recommended to mitigate the emergence and spread of resistant and pathogenic *E. coli* in the dairy processing environment.

## Figures and Tables

**Table 1:** *E. coli* isolated from raw milk and raw milk dairy products.

Sample Type	Number of Samples		Commensal *E. coli* (%)	F/Q Resistant	ESBL/AmpC Producer	Carba Producer	Total *E. coli* (%)
	Tested	Negative					
RM-NRCP	87	65	22 (25.3)	3 (3.4)	1 (1.1)	0 (0)	26 (29.9)
RM-FBO	38	24	14 (36.8)	0 (0)	1 (2.6)	0 (0)	15 (39.5)
RDP	14	9	5 (35.7)	1 (7.1)	0 (0)	0 (0)	6 (42.9)
Total	139	98	41 (29.5)	4 (2.88)	2 (1.4)	0 (0)	47 (33.8)

Abbreviations: RM-NRCP, raw milk collected as part of the national residue control plan of Ireland; RM-FBO: raw milk retailed as raw milk; RDP: raw milk dairy product; F/Q: Fluoroquinolone; Carba: Carbapenemase.

**Table 2:** AMR profiles of commensal *E. coli* and presumptive ESBL-/AmpC-producing and fluroquinolone-resistant *E. coli* isolates.

Source	Phenotypic AMR Profile	Commensal *E. coli*	ESBL/AmpC Producer	Fluoroquinolone Resistant
RM-NRCP	Amp, Chl, Cip, Nal, Smx, Tet, Tmp			2
	Amp, Ctx, Fox, Caz, Smx, Tmp		1	
	Amp, Chl, Smx, Tet, Tmp	1		
	Amp, Cip, Nal, Tet			1
	Amp, Smx, Tet, Tmp	1		
	Amp, Smx, Tmp	3		
	Fully Susceptible	17		
RM-FBO	Amp, Ctx, Caz, Chl, Fep, Gen, Smx, Tet, Tmp		1	
	Amp, Smx, Tet	1		
	Fully susceptible	13		
RDP	Cip, Nal, Smx, Tmp			1
	Amp, Tet	1		
	Fully Susceptible	4		
Total no. isolates	47	41	2	4

Abbreviations: RM-NRCP: raw milk collected as part of the national residue control plan of Ireland; RM-FBO: raw milk retailed as raw milk; RDP: raw milk dairy product; Amp: ampicillin; Chl: chloramphenicol; Cip: ciprofloxacin; Nal: nalidixic acid; Smx: sulfamethoxazole; Tet: tetracycline; Tmp: trimethoprim; Ctx: cefotaxime; Fox: cefoxitin; Caz: ceftazidime; Fep: cefepime; Gen,: gentamicin.

## Data Availability

Sequencing data produced in this study were deposited into the NCBI Sequence Read Archive (SRA) repository and are available through the BioProject accession number PRJNA1415327.

## Supplementary Materials

The following supporting information can be downloaded at: https://www.mdpi.com/article/10.3390/antibiotics15040326/s1. Supplementary Table S1. Accession numbers along with quality metrics of assemblies are detailed in Supplementary Table S1. The de novo assemblies consisted of an average of 299 contigs with an average N50 of 148,889 bp. The average coverage and genome size of the assembled reads were 148X and 5.00 Mb, respectively. Supplementary Table S2. Minimum inhibitory concentration values obtained from the 32 isolates that underwent WGS.
